# Supplementary material for: HIVprotI: an integrated web based platform for prediction and design of HIV proteins inhibitors
Source: J Cheminform. 2018 Mar 9;10:12. doi: 10.1186/s13321-018-0266-y (PMC5845081; doi:10.1186/s13321-018-0266-y)
Supplement: Supplementary file 2 — Additional file 2. Source code of HIVProtI web server. [file 13321_2018_266_MOESM2_ESM.zip › HIVprotI_Source-code/hivprotI/jsme/jme_examples/jme_example4.html]

JME Example 4


## Adding additional functionality to JME by JavaScript

Capabilities of JME may be enhanced by adding relatively simple
JavaScript code to the page containing JME.
  

### Substituent menu

This option allows fast and convenient creation of common organic
substituents. Check the JavaScript code in this page to see how the
substituent menu should be implemented.

### Molecule persistency

Several users asked for a "persistent molecule" in the JME Editor. That
means that when they visit another page and then come back, the JME
should remember the molecule recently edited. It is relatively easy to
implement this feature. It requires just to add two JavaScript
functions to the page (saveJMECookie and read JMECookie), as shown in
the source of this page. Additionally, respective event handlers must
be added into the page BODY tag.
  
Whenever the HTML page with the editor is abandoned, the
actual molecule is stored into a so called "cookie" (a small piece of
persistent information associated with this particular web page) and
when the page is revisited (even after several weeks), the molecule is
automatically restored.

|  |  |
| --- | --- |
|  | Select substituent -C(=O)OH -C(=O)OMe -OC(=O)Me -CMe3 -CF3 -CCl3 -NO2 -SO2-NH2 -NH-SO2-Me -NMe2 -C#N -C#C-Me -C#CH |

You have to enable Java in your
browser.

|  |  |
| --- | --- |
| JME Editor courtesy of Peter Ertl, Novartis | JME help |
